# *In Vivo* Amyloid-β Imaging in the APPPS1–21 Transgenic Mouse Model with a ^89^Zr-Labeled Monoclonal Antibody

**DOI:** 10.3389/fnagi.2016.00067

**Published:** 2016-03-31

**Authors:** Ann-Marie Waldron, Jens Fissers, Annemie Van Eetveldt, Bianca Van Broeck, Marc Mercken, Darrel J. Pemberton, Pieter Van Der Veken, Koen Augustyns, Jurgen Joossens, Sigrid Stroobants, Stefanie Dedeurwaerdere, Leonie Wyffels, Steven Staelens

**Affiliations:** ^1^Faculty of Medicine and Health Sciences, Molecular Imaging Center Antwerp, University of AntwerpAntwerp, Belgium; ^2^Translational Neurosciences, University of AntwerpAntwerp, Belgium; ^3^Laboratory of Medicinal Chemistry, University of AntwerpAntwerp, Belgium; ^4^Department of Neuroscience, Janssen Research and Development, A Division of Janssen Pharmaceutica NVBeerse, Belgium; ^5^Nuclear Medicine, University Hospital AntwerpAntwerp, Belgium

**Keywords:** amyloid imaging, monoclonal antibody, ^89^Zirconium, small animal imaging, Alzheimer

## Abstract

**Introduction:** The accumulation of amyloid-β is a pathological hallmark of Alzheimer’s disease and is a target for molecular imaging probes to aid in diagnosis and disease monitoring. This study evaluated the feasibility of using a radiolabeled monoclonal anti-amyloid-β antibody (JRF/AβN/25) to non-invasively assess amyloid-β burden in aged transgenic mice (APPPS1–21) with μPET imaging.

**Methods:** We investigated the antibody JRF/AβN/25 that binds to full-length Aβ. JRF/AβN/25 was radiolabeled with a [^89^Zr]-desferal chelate and intravenously injected into 12–13 month aged APPPS1–21 mice and their wild-type (WT) controls. Mice underwent *in vivo* μPET imaging at 2, 4, and 7 days post injection and were sacrificed at the end of each time point to assess brain penetrance, plaque labeling, biodistribution, and tracer stability. To confirm imaging specificity we also evaluated brain uptake of a non-amyloid targeting [^89^Zr]-labeled antibody (trastuzumab) as a negative control, additionally we performed a competitive blocking study with non-radiolabeled Df-Bz-JRF/AβN/25 and finally we assessed the possible confounding effects of blood retention.

**Results:** Voxel-wise analysis of μPET data demonstrated significant [^89^Zr]-Df-Bz-JRF/AβN/25 retention in APPPS1–21 mice at all time points investigated. With *ex vivo* measures of radioactivity, significantly higher retention of [^89^Zr]-Df-Bz-JRF/AβN/25 was found at 4 and 7 days pi in APPPS1–21 mice. Despite the observed genotypic differences, comparisons with immunohistochemistry revealed that *in vivo* plaque labeling was low. Furthermore, pre-treatment with Df-Bz-JRF/AβN/25 only partially blocked [^89^Zr]-Df-Bz-JRF/AβN/25 uptake indicative of a high contribution of non-specific binding.

**Conclusion:** Amyloid plaques were detected *in vivo* with a radiolabeled monoclonal anti-amyloid antibody. The low brain penetrance of the antibody in addition to non-specific binding prevented an accurate estimation of plaque burden. However, it should be noted that [^89^Zr]-Df-Bz-JRF/AβN/25 nevertheless demonstrated *in vivo* binding and strategies to increase brain penetrance would likely achieve better results.

## Introduction

Alzheimer’s disease (AD) is a chronic neurodegenerative disease and the leading cause of dementia in the elderly population. One of its pathological hallmarks is the accumulation of extracellular amyloid-β deposits, arising from the amyloidogenic processing of amyloid precursor protein (APP) (Selkoe, 1991). The deposition of amyloid-β is an early pathogenic event in AD, proposed to initiate 20–30 years before clinical symptoms become manifest (Bateman et al., 2012). The ability to detect and quantify amyloid-β *in vivo* could thus facilitate early diagnosis and monitoring of therapeutic responses to anti-amyloid agents (Rowe and Villemagne, 2013). Based on this premise several positron emission tomography (PET) radiotracers capable of detecting amyloid-β have been developed. While amyloid tracers such as [^11^C]-labeled Pittsburgh compound B ([^11^C]-PiB) demonstrate increased retention from prodromal through to late disease stages (Li et al., 2008; Rowe et al., 2010), they recognize only fibrillar amyloid-β, thus neglecting a wide diversity of biochemical and morphological amyloid-β subtypes. Moreover non-specific accumulation of these tracers in white matter (Fodero-Tavoletti et al., 2009) and amyloid-unrelated regions has raised concerns over their purported specificity (Kepe et al., 2012). The ability to image a wider spectrum of amyloid-β subtypes would provide greater understanding of the composition of plaques and the pharmacological effects of amyloid-β targeted therapies. However, the specificity required for this purpose is not achievable with small molecules and a more viable approach would be the use of radiolabeled epitope specific anti-amyloid antibodies.

With this objective in mind we previously evaluated a radiolabeled monoclonal mouse antibody (mAb) directed against amyloid-β for use as an *in vivo* imaging agent (Fissers et al., 2016). JRF/AβN/25 is an N-terminal end-specific antibody directed against the first seven amino acids of human Aβ1–40/42 (Vandermeeren et al., 2001; Mathews et al., 2002) and thus recognizes full length Aβ peptides. With the expected low brain permeability of the antibody, the long-lived radioisotope ^89^Zr (T ½ = 78.41 h) was chosen as a suitable radiolabel that would accommodate the long biological half-life of antibodies and possible accumulation of antibody over time on its target. In our previous work (Fissers et al., 2016), ^89^Zr was attached to JRF/AβN/25 using the bifunctional chelator desferal. Using an *in vitro* saturation assay we demonstrated a nanomolar affinity (*Kd* = 0.85 nM) of [^89^Zr]-Df-Bz-JRF/AβN/25 against Aβ1–40 in addition to low non-specific binding. Under *in vitro* conditions autoradiographs showed binding of [^89^Zr]-Df-Bz-JRF/AβN/25 to amyloid-β plaques in the brain of an APPPS1–21 mouse. Lastly, *in vivo* tests in healthy mice indicated tracer stability, a favorable biodistribution and some modest brain penetrance (Fissers et al., 2016). Given these promising results this study aimed to investigate whether the radiolabeled antibody could be used in conjunction with micro-PET (μPET) imaging to quantify amyloid-β burden. We performed a cross-sectional investigation of tracer uptake over a 7 days period in aged transgenic APPPS1–21 and wild-type (WT) mice. To cross-validate *in vivo* radioactivity measures, brain uptake was also measured with γ-counting and autoradiography. As a gold standard evaluation, plaques labeled *in vivo* were assessed with immunohistochemistry and compared to total plaque load to provide a measure of sensitivity. To assess tracer specificity several additional experiments were conducted. These involved a negative control study with the non-amyloid targeted [^89^Zr]-trastuzumab antibody (Slamon et al., 2001), a competitive blocking study and lastly a study to assess possible confounding effects of blood radioactivity.

## Materials and Methods

### Animals

An overview of the animals involved in each experiment and the procedures performed are shown in **Table 1**. Female APPPS1–21 mice and their littermate controls were received in kind from Janssen Pharmaceutica (Beerse, Belgium). APPPS1–21 mice overexpress human mutant amyloid precursor protein (hAPP695swe) and presenilin -1 (L166P) under the control of the neuron specific Thy-1 promoter on a C57BL/6J background and develop insoluble amyloid-β deposition in the cortex as of 6 weeks of age (Radde et al., 2006).

**Table 1:** Overview of study design.

Study	Antibody	Animals		Age (months)	Time point (days pi)	Procedures				
		WT	APPPS1–21			μPET	ARG	Biodistribution	IHC	Plasma stability
Proof of concept	[^89^Zr]-Df-Bz-JRF/AβN/25	18	18	12–13	2, 4,7	√	√	√	√	√
Negative control	[^89^Zr]-trastuzumab	3	3	12–13	4	√	√	√	√	–
Blocking	[^89^Zr]-Df-Bz-JRF/AβN/25	–	6	12–13	4	√	√	√	√	–
Blood contribution	[^89^Zr]-Df-Bz-JRF/AβN/25	3	3	12–13	4	–	√	Brain only	√	–

The animals were kept under environmentally controlled conditions (12 h light/dark cycle, 20–24°C and 40–70% relative humidity) in individually ventilated cages with food and water ad libitum. Animals were group-housed and received environmental enrichment. The study protocol was approved by the local Animal Experimental Ethical Committee of the University of Antwerp, Belgium (2012–2025) where the *in vivo* experiments were performed. All animal studies were ethically reviewed and carried out in accordance with European Directive 86/609/EEC Welfare and Treatment of Animals.

### [^89^Zr]-Labeling of the Antibody and Stability Measures

JRF/AβN/25 was radiolabeled with ^89^Zr using a desferal chelator as described previously (Fissers et al., 2016). [^89^Zr]-trastuzumab was received in kind from BV Cyclotron (Amsterdam, The Netherlands). Plasma was obtained from blood by centrifugation and analyzed by SEC-HPLC and radio-iTLC for the presence of radiometabolites as described previously (Fissers et al., 2016).

### *In Vivo* Imaging with μPET and *Ex Vivo* Biodistribution

In the *proof of concept, negative control*, and *blood contribution* studies (**Table 1**), mice received a lateral tail vein injection of 133 μg in maximum 200 μl volume of [^89^Zr]-Df-Bz-JRF/AβN/25 or [^89^Zr]-trastuzumab. In the *blocking study* mice were injected with modified unlabeled Df-Bz-JRF/AβN/25 (400 μg in volume of 100 μl) 2 h prior to receiving the radiolabeled mAb (volume of 150 μl). All μPET scans were static acquisitions. To account for variation in injected dose and time difference between scanning, the length of μPET acquisitions was determined on the basis of equivalent count rates. Anesthesia was induced by inhalation of 2% isoflurane (5% for induction, 2% for maintenance) supplemented with oxygen. All μPET data was reconstructed as described previously (Waldron A. et al., 2015).

Volume-of-interest (VOI) and voxel-wise analysis (statistical parametric mapping) methods were performed on reconstructed images using PMOD v3.3 (PMOD technologies, Switzerland) and SPM8 (Wellcome Trust Centre, UK). Individual PET images were spatially normalized into the space of a predefined mouse brain template (Mirrione et al., 2007), masked to remove extracerebral activity and then expressed in percent injected dose (tissue uptake_[kBq/cc]_/ injected dose_[kBq]_^∗^ 100). For VOI analysis, images were subsequently co-registered with a predefined mouse brain VOI template aligned with the aforementioned Mirrione CT/MRI atlas and tracer uptake values were extracted for each delineated VOI. With this approach regional values are quantified as the average uptake over the total number of voxels in a VOI. For voxel-wise analysis of tracer uptake between genotypes a two-sample unpaired *t*-test was applied to images, with a significance threshold of *p* < 0.01. To control for errors at a reasonable level we additionally set an extent threshold of 100 voxels (48 mm^3^). In this instance a voxel is only considered active if it belongs to a cluster of 100 contiguous active voxels. Quantification of this voxel-wise analysis was investigated by calculating the number of significant voxels in a VOI [CT/MRI template (Mirrione et al., 2007)] relative to the total number of voxels (% sign) in that region. Images were smoothed with a Gaussian filter (0.5 mm) for visualization and are overlaid on a T_2_-weighted MRI template for anatomical reference.

Subsequent to scanning, mice were kept under isoflurane anesthesia and a cardiac puncture was performed to collect blood. Thereafter mice were sacrificed by cervical dislocation and all organs were removed, rinsed in buffer, and blotted dry. The brain was hemisected with the left hemisphere snap frozen in chilled 2-methyl-butane for autoradiography and the right hemisphere fixed in formaldehyde. Radioactivity in the samples was measured using an automatic γ-counter. The uptake of radioactivity in blood, right brain hemisphere and peripheral organs was expressed as percentage of the injected dose per gram of tissue plus or minus the standard deviation (%ID/g ± SD).

In the *blood contribution study* mice were anesthetized with an intraperitoneal injection of Nembutal (200 mg/kg) and perfused transcardially with ice-cold 0.1 M phosphate buffer saline (PBS) to remove blood. Thereafter brain tissue was processed as described above.

### *Ex Vivo* Validation by Autoradiography and Immunohistochemistry

Sagittal brain slices (20 μm) were obtained through cryostat sectioning (Leica) of the frozen left brain hemisphere. After air-drying at 37°C, the sections were co-exposed with a ^14^C standard on a phosphor imaging plate (Fujifilm BAS IP MS 2025) for 72 h. Following exposure, plates were read using a Fuji Phosphorimager system (FLA-700). Whole brain slice radioactivity concentrations were calculated through manual region of interest delineation (ImageJ). Radioactivity concentration was quantified through interpolation of measured values in a ^14^C standard curve (Van Eetveldt and Dedeurwaerdere, under review) and then corrected for injected dose and radioactive decay.

Immunohistochemistry was performed on slices from the left hemisphere (fresh frozen) according to two protocols. The objective of the first protocol was to assess *in vivo* antigen labeling, in this way the injected [^89^Zr]-labeled antibody acted as a primary antibody. Brain slices were thus incubated with only secondary antibody to stain areas in which injected antibody had bound. In the second protocol total plaque load was stained for by applying exogenous primary antibody on brain slices and then with secondary antibody as above. Comparison of the results obtained with these protocols allowed the amount of antigen captured through *in vivo* uptake relative to the true amount of antigen available to be determined. Staining was performed on a Ventana Discovery autostainer using a chromomap detection kit. Briefly sections were rinsed in wash buffer then incubated in primary antibody diluted in PBS (JRF/AβN/25, 1/500) at 37°C for 28 min. Sections were rinsed again in wash buffer and then fixed in NBF (neutral buffered formalin) for 4 min. Secondary anti-mouse antibody conjugated to HRP (1/50 dilution in PBS) was applied for 28 min and DAB was used as a chromogen for visualization of the staining. Finally sections were counterstained with hematoxylin, mounted, and coverslipped.

Brain tissue from the right brain hemisphere of a subset of animals at 4 days pi also underwent staining for total plaque load. This tissue was preferentially used for quantification due to reduced non-specific background obtained with this protocol. After γ-counting, brain tissue was fixed, paraffin-embedded, and cut into 5-μm coronal sections. De-waxed and rehydrated sections underwent tissue depigmentation in potassium permanganate, oxalic acid and water and were subsequently pretreated with 70% formic acid for 10 min for epitope retrieval. Endogenous peroxidase activity was blocked by rinsing in 3% hydrogen peroxide for 5 min. Thereafter, sections were incubated with mouse anti-amyloid primary antibody (JRF/AβN/25 1:10,000 1 h) and processed as described above. Virtual images were acquired using a Mirax Digital Slide Scanner (Zeiss), and image analysis was performed using the Definiens analysis software package v1.5. Regions of interests (ROIs) were manually delineated in accordance with Franklin and Paxinos atlas (Franklin and Paxinos, 1997), and for each ROI, the percentage of DAB-labeled area was calculated.

## Results

### [^89^Zr]-Df-Bz-JRF/AβN/25 Demonstrates Stable Pharmacokinetics *In Vivo*

The average uptake of [^89^Zr]-Df-Bz-JRF/AβN/25 in APPPS1–21 and WT mice in selected organs are shown in **Table 2**. There were high circulating levels of [^89^Zr]-Df-Bz-JRF/AβN/25 present in blood at 2 days pi with 9.36 ± 1.38%ID/g in APPPS1–21 mice and 8.41 ± 2.23%ID/g in WT. Blood activity gradually decreased to 4.62 ± 1.76%ID/g and 5.08 ± 1.11%ID/g at 7 days pi, respectively. Liver uptake was high at all time points investigated and is indicative of hepatobiliary clearance. There was a notable accumulation of radioactivity in skull bone with highest levels of uptake at 7 days pi, 5.84 ± 3.19%ID/g in APPPS1–21 and 6.43 ± 1.76%ID/g in WT. Plasma stability of [^89^Zr]-Df-Bz-JRF/AβN/25 was high at all time points as measured by both HPLC and iTLC, with the latter method generally showing higher values. By 7 days pi levels of intact [^89^Zr]-Df- Bz-JRF/AβN/25 were 96.37 ± 1.76% in WT and 94.73 ± 1.28% in APPPS1–21 plasma, respectively, as quantified by iTLC. Stability results from earlier time points and from HPLC measures can be found in Supplementary Table S1.

**Table 2:** Selected *ex vivo* biodistribution data of [^89^Zr]-Df- Bz-JRF/AβN/25 at different time points after intravenous injection in APPPS1–21 and WT mice.

Genotype	Time (d)	Blood	Heart	Lungs	Liver	Spleen	Kidneys	Muscle	Skull bone
APPPS1–21	2	9.36 ± 1.38	2.55 ± 0.46	3.72 ± 0.63	6.91 ± 1.92	6.18 ± 1.53	3.93 ± 0.52	0.52 ± 0.15	3.92 ± 3.09
	4	7.19 ± 1.3	2.17 ± 0.41	4.04 ± 1.12	8.29 ± 1.67	6.76 ± 0.73	3.9 ± 0.46	0.44 ± 0.09	3.4 ± 0.94
	7	4.62 ± 1.76	1.37 ± 0.49	2.17 ± 0.73	6.88 ± 1.22	7.07 ± 2.91	2.59 ± 0.73	0.43 ± 0.13	5.84 ± 3.19
WT	2	8.41 ± 2.23	2.43 ± 0.67	3.19 ± 0.96	5.27 ± 0.96	4.82 ± 1.25	3.59 ± 1.12	0.51 ± 0.19	5.23 ± 2.69
	4	7.94 ± 1.65	2.07 ± 0.35	3.59 ± 0.57	8.15 ± 1.66	6.89 ± 1.74	3.51 ± 0.3	0.45 ± 0.08	4.78 ± 2.74
	7	5.08 ± 1.11	1.6 ± 0.34	2.5 ± 0.46	10.63 ± 2.44	8.21 ± 2.38	2.94 ± 0.35	0.37 ± 0.09	6.43 ± 1.76

*Data is represented as the average percent of injected activity per gram tissue ± SD.*

### Transgenic Mice Demonstrate Increased Brain Uptake of [^89^Zr]-Df-Bz-JRF/AβN/25

**FIGURE 1:**
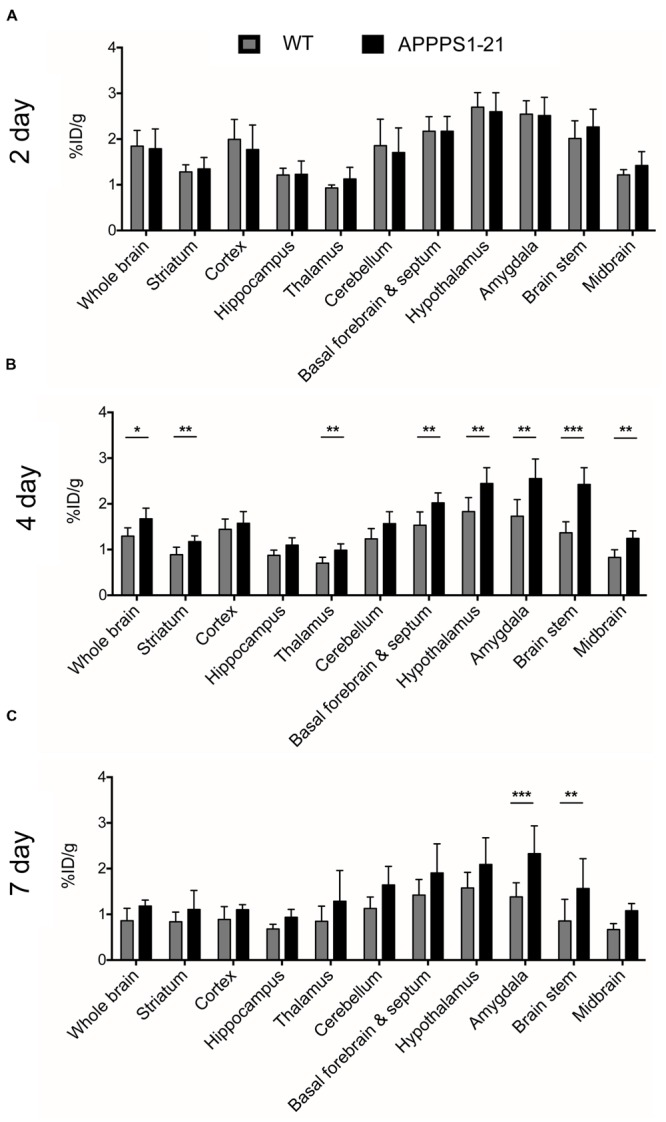
**μPET imaging demonstrates increased brain uptake of [^89^Zr]-Df-Bz-JRF/AβN/25 in APPPS1–21 mice in comparison to WT.** Mice were injected with 133 μg of [^89^Zr]-Df-Bz-JRF/AβN/25 and brain uptake was measured by μPET scanning and quantified as the %ID/g. The graphs show the VOI analysis of [^89^Zr]-Df-Bz-JRF/AβN/25 retention in WT and APPPS1–21 mice at **(A)** 2 days pi, **(B)** 4 days pi, and **(C)** 7 days pi. Significant genotype differences in retention were observed at 4 and 7 days pi. Data is presented as mean + SD. Student’s *t*-test, ^∗∗∗^*p* < 0.001, ^∗∗^*p* < 0.01, ^∗^*p* < 0.05.

**FIGURE 2:**
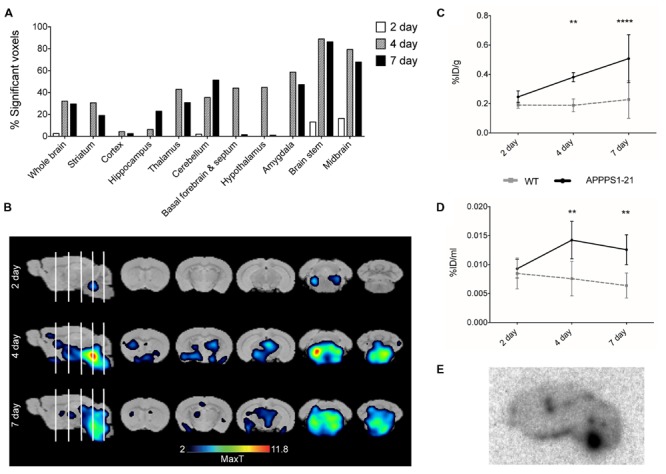
**Voxel-wise analysis of the μPET data and *ex vivo* examination of brain tissue confirms higher accumulation of [^89^Zr]-Df-Bz-JRF/AβN/25 in APPPS1–21 mice.** Graph **(A)** shows voxel-wise analysis, data is presented as the percent of each brain region that was significantly changed (increase). This data is illustrated in image **(B)**, the statistical T-maps represent areas of significant increases. After imaging mice were sacrificed and brain radioactivity was measured *ex vivo* by **(C)** γ–counting and **(D)** autoradiography. Data is presented as the average + SD. **(E)** Representative autoradiograph of [^89^Zr]-Df-Bz-JRF/AβN/25 uptake at 4 days pi in an APPPS1–21 mouse. Student’s *t*-test ^∗∗∗∗^*p* < 0.0001, ^∗∗^*p* < 0.01.

**Figure 1** displays the results from VOI analysis of the PET imaging. We used unpaired Student’s *t*-tests (corrected for multiple comparisons) to compare [^89^Zr]-Df-Bz-JRF/AβN/25 retention between brain regions with this analysis. At 2 days pi no clear differences were observed between WT and APPPS1–21 mice (**Figure 1A**). At 4 days pi, APPPS1–21 mice demonstrated increased [^89^Zr]-Df-Bz-JRF/AβN/25 retention in all brain regions investigated (**Figure 1B**). The most significant differences were found in the brain stem (*p* = 0.00015) followed by the midbrain (*p* = 0.0015) and thalamus (*p* = 0.0045). By 7 days pi significant differences remained only in the brain stem (*p* = 0.0021) and amygdala (*p* = 0.000057) (**Figure 1C**). With a more sensitive voxel-wise analysis (**Figure 2A**), significant differences in [^89^Zr]-Df-Bz-JRF/AβN/25 retention between genotypes was observed already at 2 days pi in the midbrain (16.3%) and brain stem (13%). By 4 days pi, significant voxels increased substantially in the brain stem (89%), midbrain (79.3%), amygdala (58.7%), hypothalamus (44.7%), and basal forebrain and septum (44%). While the percent of significant voxels in the whole brain remained relatively constant between 4 and 7 days pi (32.1 and 29.6%, respectively), regionally there were less significant voxels in the basal forebrain and septum (from 44% to 1.5%) and hypothalamus (44.7 to 1%) and an increase in the cerebellum (from 35.4 to 51.3%). **Figure 2B** illustrates the localization of significant voxels on an anatomical brain template.

Subsequent to μPET scanning, brain radioactivity was measured *ex vivo* by γ-counting. **Figure 2C** shows the brain uptake values of [^89^Zr]-Df-Bz-JRF/AβN/25 in both WT and APPPS1–21 mice for all time points as measured by γ-counting. With this method APPPS1–21 mice demonstrated a progressive uptake of [^89^Zr]-Df-Bz-JRF/AβN/25, reaching peak uptake values at 7 days pi (0.51 ± 0.16%ID/g). Retention in WT remained constant over time. Using a 2-way ANOVA with genotype and time as co-factors a significant effect was found for both factors for [^89^Zr]-Df-Bz-JRF/AβN/25 uptake [*F*_(1,29)_ = 32.68, *p* < 0.0001 for genotype and *F*_(2,29)_ = 8.183, *p* = 0.0015 for uptake time]. Using unpaired Student’s *t*-tests (corrected for multiple comparisons) between WT and APPPS1–21 uptake at each time point, significantly higher retention of [^89^Zr]-Df-Bz-JRF/AβN/25 was found at 4 (*p* < 0.01) and 7 days pi (*p* < 0.0001). With *ex vivo* autoradiography a significant genotype effect only was observed for [^89^Zr]-Df-JRF/AβN/25 retention [*F*_(1,24)_ = 20.73, *p* < 0.0001]. In contrast to the trend observed with γ-counting, uptake of [^89^Zr]-Df-JRF/AβN/25 was highest at 4 days pi (0.014 ± 0.003%ID/ml) (**Figure 2D**). Retention of [^89^Zr]-Df-JRF/AβN/25 in APPPS1–21 mice was significantly higher than in WT at 4 and 7 days pi (both *p* < 0.01). Localization of cerebral radioactivity at 4 days pi in APPPS1–21 mice is shown by a representative autoradiogram in **Figure 2E**.

### [^89^Zr]-Df-Bz-JRF/AβN/25 Labels Plaques to a Low Extent after i.v Injection

**FIGURE 3:**
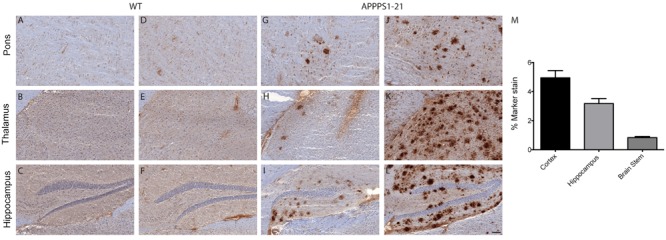
**Immunohistochemistry demonstrates that [^89^Zr]-Df-Bz-JRF/AβN/25 labels plaques to a low extent after i.v injection.** Sagittal brain slices (20 μm) were cut and the sections were stained for amyloid-β. Images **(A–C,G–I)** represent *in vivo* labeling arising from i.v injections of [^89^Zr]-Df-Bz-JRF/AβN/25 and are compared to adjacent slices that were stained for total plaque load **(D–F,J–L)**. (Scale bar = 100 μm). Graph **(M)** depicts quantification of amyloid-β staining in the cortex, hippocampus and brain stem. Data is shown as the mean + SD.

The entry of [^89^Zr]-labeled antibodies into the brain was further evaluated *ex vivo* by immunostaining fresh-frozen brain sections with secondary antibody (**Figure 3**). APPPS1–21 mice displayed plaque-staining arising from the i.v injections of [^89^Zr]-Df-Bz-JRF/AβN/25 (**Figures 3G–I**). However, immunostained plaques were sparse and predominantly located in the hippocampal region, the mammillary nucleus and the pons. Adjacent brain sections were analyzed for total antigen by exogenous addition of primary antibody and subsequent standard immunohistochemical procedures. This analysis revealed widespread plaque load (**Figures 3J–L**). Plaques were not observed in WT mice (**Figures 3A–F**). We quantified plaque load in the cortex, hippocampus, and brain stem of a subset of animals (three TG and three WT) at 4 days pi using fixed tissue from the right hemisphere. The cortex and hippocampus demonstrated plaque load (4.95 ± 0.5% and 3.18 ± 0.35% marker stain, respectively) while plaque staining was minimal in the brain stem (0.83 ± 0.08% marker stain) (**Figure 3M**).

### The Effects of Increased Blood–Brain Barrier Permeability and Blood Retention of [^89^Zr]-Df-Bz-JRF/AβN/25 are Insignificant in APPPS1–21 Mice

**FIGURE 4:**
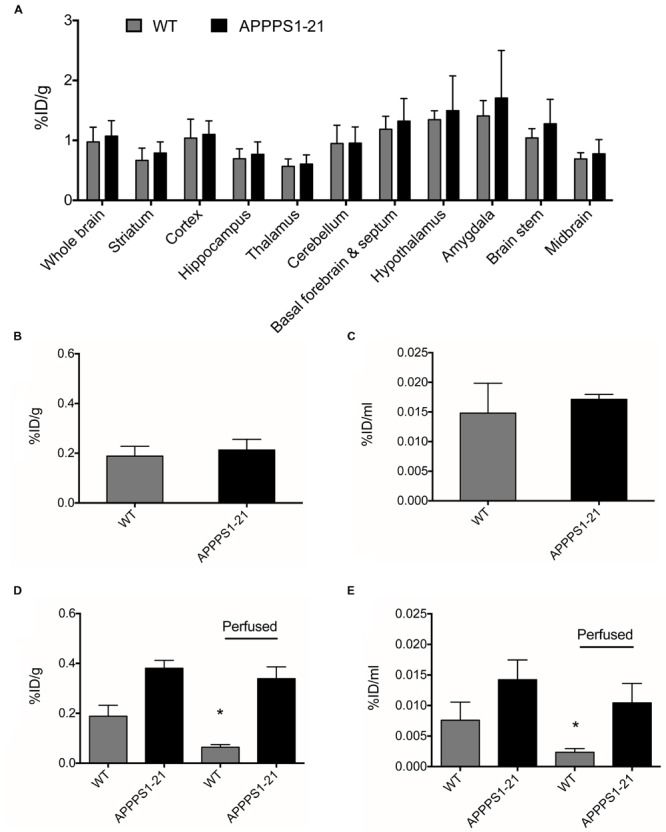
**APPPS1–21 mice did not exhibit significant uptake of a non-amyloid targeted antibody in comparison to WT mice.** Mice received an i.v injection of 133 μg of [^89^Zr]-trastuzumab and were underwent μPET scanning at 4 days pi. Graph **(A)** depicts VOI analysis of [^89^Zr]-trastuzumab retention in WT and APPPS1–21 mice, data is presented as mean + SD. After scanning, mice were sacrificed and brain radioactivity of [^89^Zr]-trastuzumab was measured by **(B)** γ–counting and **(C)** autoradiography. No significant differences in cerebral [^89^Zr]-trastuzumab retention between genotypes were observed with either *in vivo* or *ex vivo* methods. We compared brain retention of [^89^Zr]-Df-Bz-JRF/AβN/25 in APPPS1–21 with and without transcardial perfusion by **(D)** γ–counting and **(E)** autoradiography, data is presented as mean + SD. Student’s *t*-test ^∗^*p* < 0.05. Retention of [^89^Zr]-Df-Bz-JRF/AβN/25 was significantly reduced in perfused WT but not perfused APPPS1–21 mice.

Cerebral uptake of a non-amyloid targeted [^89^Zr]-labeled antibody was investigated as a negative control. No significant differences in cerebral [^89^Zr]-trastuzumab retention between genotypes were observed with any of the employed methods (**Figures 4A–C**). In comparison to cerebral retention of [^89^Zr]-Df-Bz-JRF/AβN/25 in APPPS1–21 mice, [^89^Zr]-trastuzumab retention was 1.8 times lower (Supplementary Table S4). Blood and peripheral organ retention of [^89^Zr]-trastuzumab was, however, similar to that demonstrated for [^89^Zr]-Df-Bz-JRF/AβN/25 at 4 days pi (Supplementary Table S2).

To further investigate the *in vivo* specificity of [^89^Zr]-Df-Bz-JRF/AβN/25 we assessed the contribution of cerebral blood to the measurements of brain [^89^Zr]-Df-Bz-JRF/AβN/25 retention. After tissue perfusion, brain retention of [^89^Zr]-Df-Bz-JRF/AβN/25 significantly decreased (*p* = 0.0238) by 66% in WT mice and non-significantly decreased by 11% in APPPS1–21 mice (**Figure 4D**) as measured by γ-counting. Autoradiography demonstrated a similar trend with WT showing a reduction of 67% (*p* = 0.0238) and APPPS1–21 demonstrating a non-significant decrease of 27% (*p* = 0.1736) (**Figure 4E**). Immunohistochemistry confirmed that plaques in perfused APPPS1–21 mice were visible in the same brain regions as for non-perfused mice.

### Increased Brain Retention of [^89^Zr]-Df-Bz-JRF/AβN/25 is Mediated in Part by Non-specific Effects in APPPS1–21 Mice

**FIGURE 5:**
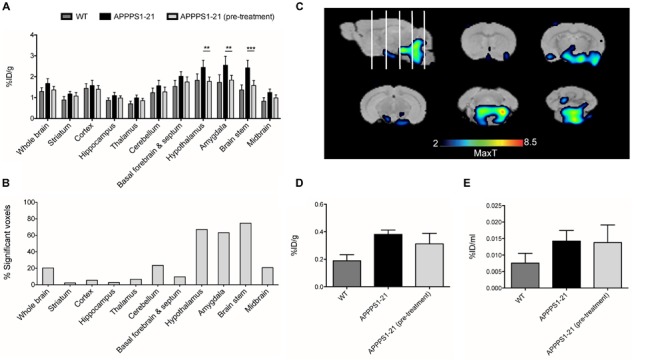
**Pre-treatment with non-labeled Df-Bz-JRF/AβN/25 only partially blocks [^89^Zr]-Df-Bz-JRF/AβN/25 uptake in APPPS1–21 mice.** APPPS1–21 mice were treated with 400 μg of non-labeled Df-Bz-JRF/AβN/25 2 h prior to injection of [^89^Zr]-Df-Bz-JRF/AβN/25. Brain retention was assessed at 4 days pi. Graph **(A)** depicts VOI analysis of the μPET imaging, data is presented as mean + SD. Graph **(B)** shows voxel-wise analysis, data is presented as the % of each brain region that was significantly changed (decrease). Image **(C)** is statistical T-maps depicting regions of significantly reduced tracer retention in pre-treated versus untreated APPPS1–21 mice. Radioactivity was measured *ex vivo* by **(D)** γ–counting and **(E)** autoradiography. Data is presented as the average ± SD. Student’s *t*-test, ^∗∗∗^*p* < 0.001, ^∗∗^*p* < 0.01.

Lastly, we conducted a competitive *blocking study* (**Figure 5**). Pre-administration of 400 μg unlabeled Df-Bz-JRF/AβN/25 to APPPS1–21 mice did not alter blood or organ retention of [^89^Zr]-Df-Bz-JRF/AβN/25 (Supplementary Table S3). VOI analysis of the μPET imaging data showed significantly reduced [^89^Zr]-Df-Bz-JRF/AβN/25 retention in pre-treated APPPS1–21 mice in the brain stem (*p* = 0.0008), hypothalamus (*p* = 0.0023), and amygdala (0.0048), when compared to un-treated APPPS1–21 at the same 4 days pi time point (**Figure 5A**). With voxel-wise analysis we similarly showed the greatest significant decreases between pre-treated and untreated APPPS1–21 mice in these regions and additionally demonstrated appreciable differences in the cerebellum (23.5%) and midbrain (20.8%) (**Figures 5B,C**). With *ex vivo* measures, pre-treatment non-significantly reduced brain retention in APPPS1–21 mice by 18 and 3.17% as measured by γ-counting and autoradiography, respectively, (**Figures 5D,E**). Immunohistochemistry confirmed the same regional pattern of staining for mice with or without pre-treatment.

## Discussion

This study aimed to investigate the potential of a radiolabeled monoclonal anti-amyloid-β antibody as an *in vivo* imaging agent. [^89^Zr]-Df-Bz-JRF/AβN/25 demonstrated brain penetrance after intravenous injection and showed significantly higher retention in APPPS1–21 mice in comparison to their WT littermates. Despite these increases, immunohistochemistry revealed sparse plaque labeling that was confined to a limited number of brain regions in APPPS1–21 mice. Furthermore plaque staining was not confirmed in all regions that demonstrated increased *in vivo* retention with μPET imaging which we attribute to possible non-specific binding of the mAb in addition to the resolution limitations of μPET imaging. The low brain penetrance and non-specific binding of the mAb results in a low signal to noise ratio and prevents an accurate estimation of total amyloid-β load thus precluding its use as a biomarker with the current methods.

The entry of full-length antibodies across the blood–brain barrier is known to be low and has been previously estimated as 0.11% of the injected dose (Banks et al., 2002). High blood concentrations and long systemic circulation can promote greater uptake (Misra et al., 2003). Our previous study demonstrated high blood radioactivity of [^89^Zr]-Df-Bz-JRF/AβN/25 until 2 days pi (Fissers et al., 2016) and by prolonging the uptake time to 7 days we aimed at enhancing specific accumulation of the antibody in plaque rich brain areas and additionally allow non-specific wash-out of the tracer. Blood levels of [^89^Zr]-Df-Bz-JRF/AβN/25 indeed remained relatively high up to 7 days pi and as anticipated the tracer demonstrated the most significant uptake in brain at later time points due to the better contrast provided by a higher retention in transgenic mice and a lower retention in WT. Using a [^125^I]-labeled antibody directed against Aβ protofibrils, Magnusson et al previously demonstrated the highest tracer retention in transgenic mice at 3 days pi in comparison to earlier 1 day and 3 h time points (Magnusson et al., 2013). However, even at the later time points investigated here, levels of brain retention were still too low in terms of that expected for useful radiotracers. While a brain uptake of 1%ID/g of the 6E10 anti-amyloid antibody in transgenic mice has been previously reported (McLean et al., 2012b), lower uptake values similar to those demonstrated here are more prevalent (Banks et al., 2002; Magnusson et al., 2013). Whether brain uptake of [^89^Zr]-Df-Bz-JRF/AβN/25 would continue to increase as a function of time is unknown. However, possible reductions in tracer stability and radioactivity levels (and thus image quality) over time would negate any benefits of further brain uptake. While the long half-life of ^89^Zr allowed for prolonged uptake and imaging, our biodistribution studies revealed high bone uptake. Metabolism resulting in dissociation of the [^89^Zr]-desferal chelate from the antibody and complete release of the ^89^Zr cation is the likely cause of this radioactivity. Although the plasma stability studies demonstrated a high percent of intact tracer and minimal free ^89^Zr, this may be an inaccurate estimation as free ^89^Zr is avidly sequestered by bone thus removing it from the circulation. Such high bone uptake with ^89^Zr-labeled antibodies has been previously reported in rodents with biodistribution studies showing up to 10% ID/g activity. Given the low spatial resolution of μPET scanners and the small size of the mouse brain the effect of spillover from skull bone radioactivity on the accurate quantification of cerebral uptake is a pressing issue for sensitivity. In terms of translational relevance, these mechanisms are of less importance to human imaging studies where metabolism of the [^89^Zr]-desferal chelate is considerably less extensive.

Given the low tracer amounts, [^89^Zr]-Df-Bz-JRF/AβN/25 could not accurately quantify total amyloid-β burden and yielded an *in vivo* cerebral retention pattern which did not correspond entirely to regions with known high plaque load. Immunohistochemistry for *in vivo* plaque labeling revealed targeting of [^89^Zr]-Df-Bz-JRF/AβN/25 mainly to the hippocampus and pons with minimal plaque labeling observed in frontal brain regions, areas in which the highest plaque load is present (Radde et al., 2006; Waldron A.-M. et al., 2015). This discrepancy can be seen when comparing the μPET data at 4 days pi (**Figure 1B**) to the immunohistochemistry quantification at the same time point (**Figure 3M**). From the imaging data, the brain stem has significantly higher uptake of [^89^Zr]-Df-Bz-JRF/AβN/25 than WT and a high uptake in comparison to other brain regions such as the cortex and hippocampus in the TG mice. However, *ex vivo* quantification of plaque load demonstrated minimal plaque load in the brain stem and a relatively high plaque load in the cortex and hippocampus. With the high abundance of antigen available in these aged APPPS1–21 mice it is likely that the low amounts of radiolabeled antibody become rapidly sequestered near their point of entry. In addition to this effect, the large size of the antibody may also hinder diffusion within the parenchyma (McLean et al., 2012a). Thus given the localization of staining and radioactivity around the third and fourth ventricles we hypothesize entry of the antibody through circumventricular regions. Magnusson et al. (2013) similarly noted localization of an anti-amyloid antibody to the ventricles, specifically the choroid plexus. The mechanisms accounting for brain uptake are unknown and could not be delineated by our current methods.

The cerebral levels of radioactivity in APPPS1–21 mice appear to overestimate specific mAb retention when compared with the low amount of plaque labeling observed with immunohistochemistry. To probe possible non-specific effects we conducted a number of further experiments. There is evidence to suggest that the BBB is deficient in AD patients and transgenic AD mouse models and that this alteration may allow antibodies to infiltrate the brain (Kumar-Singh et al., 2005; Erickson and Banks, 2013). While this factor may be necessary for brain antibody imaging it is also a potential confound whereby an enhanced BBB permeability in APPPS1–21 mice versus WT mice would result in higher tracer retention unrelated to amyloid-binding. To investigate such a mechanism we performed a negative control study with [^89^Zr]-trastuzumab, a humanized monoclonal antibody targeted against the herceptin receptor (Slamon et al., 2001). In comparison to WT, APPPS1–21 mice demonstrated non-significantly increased retention of [^89^Zr]-trastuzumab with both *in vivo* and *ex vivo* techniques suggesting a very modest influence of BBB integrity on mAb uptake in APPPS1–21 mice.

[^89^Zr]-Df-Bz-JRF/AβN/25 is not plaque-specific and can additionally bind to soluble amyloid-β present in blood and brain. To investigate the contribution of blood-bound [^89^Zr]-Df-Bz-JRF/AβN/25 we measured cerebral radioactivity after removal of the blood fraction through transcardial perfusion. By comparing to the previously investigated [^89^Zr]-Df-Bz-JRF/AβN/25 cohort we found brain radioactivity to be reduced by 11–27% in APPPS1–21 and 66–67% in WT after perfusion. Thus while blood retention may account for some of the radioactivity signal, it appears that tissue retention is the predominant source in APPPS1–21 mice. This tissue source encompasses both specific binding to plaques in addition to non-specific binding. To assess non-specific tissue binding a competitive *blocking study* with non-labeled Df-Bz-JRF/AβN/25 was performed. With this we demonstrated that cerebral retention of [^89^Zr]-Df-Bz-JRF/AβN/25 could be blocked to a maximum of 18% in APPPS1–21 mice, independent of changes in peripheral biodistribution. This modest reduction points to a possible contribution of non-specific tissue binding of [^89^Zr]-Df-Bz-JRF/AβN/25. However, incomplete blocking may also have resulted from the short uptake period (2 h) of the non-labeled antibody. It should be noted that the decreased uptake after pre-treatment was not confirmed by autoradiography. The main disadvantage with this technique is the requirement for brain sectioning into 2D sections which naturally results in the loss of 3D spatial consistency of the brain. In this study we analyzed triplicate sagittal brain sections (20 μm thick) for a representative sampling of brain radioactivity levels. The small volume analyzed contrasts with the whole 3D volumes measured by uPET imaging and likely accounts for the discrepancy between measurements and the apparent lower sensitivity.

A number of methods to circumvent the BBB have been employed to deliver antibodies to the brain and such techniques would likely improve the plaque detecting abilities of [^89^Zr]-Df-Bz-JRF/AβN/25. Disruption of the BBB by focused ultrasound (Jordão et al., 2010) or hyperosmotic solutions (McLean et al., 2012b) have shown increased targeting of antibodies to the brain. While these methods are valuable for proof-of-concept, mechanical and chemical BBB disruption are not clinically translatable. More viable methods have focused on antibody engineering to produce fragments of lower molecular weight (Poduslo et al., 2007) in addition to biochemical modification with polymers to enhance brain permeability (Poduslo et al., 2007; McLean et al., 2012b). Alternatively, receptor-mediated transcytosis approaches that exploit the insulin-like growth factor and transferrin receptors (Bien-Ly et al., 2011) could be employed to increase brain targeting.

## Conclusion

In conclusion, the current results suggest limited utility of [^89^Zr]-Df-Bz-JRF/AβN/25 as amyloid imaging agent with the current methods. However, it should be noted that [^89^Zr]-Df-Bz-JRF/AβN/25 nevertheless demonstrated *in vivo* plaque binding and strategies to increase brain penetrance would likely achieve better results.

## Author Contributions

AMW executed the experimental design, generated, and analyzed the imaging and biodistribution data and drafted the manuscript. JF produced the radiolabeled antibody and performed and analyzed stability measures. AVE processed and analyzed brain tissue for autoradiography and immunohistochemistry. All authors contributed to the conception of the study, coordinated the experimental design and contributed to the writing and proofreading of the final manuscript. All authors approved the final manuscript and agree to be accountable for all aspects of the work.

## Conflict of Interest Statement

The authors declare that the research was conducted in the absence of any commercial or financial relationships that could be construed as a potential conflict of interest.
