# Supplementary material for: In Vivo Amyloid-β Imaging in the APPPS1–21 Transgenic Mouse Model with a 89Zr-Labeled Monoclonal Antibody
Source: Front Aging Neurosci. 2016 Mar 31;8:67. doi: 10.3389/fnagi.2016.00067 (PMC4815004; doi:10.3389/fnagi.2016.00067)
Supplement: Supplementary file 1 [file Table_1.DOCX]

***Supplementary Material***

***In vivo* amyloid-β imaging in the APPPS1-21 transgenic mouse model with a ^89^Zr- labeled monoclonal antibody.**

Ann-Marie Waldron^1,2^, Jens Fissers^1^, Annemie van Eetveldt^2^, Bianca Van Broeck^3^, Marc Mercken^3^, Darrel J. Pemberton^3^, Pieter Van Der Veken^4^, Koen Augustyns^4^, Jurgen Joossens^4^, Sigrid Stroobants^5^, Stefanie Dedeurwaerdere^2^, Leonie wyffels^1,5^, Steven Staelens^1^*.

**Corresponding author:** Steven Staelens, Molecular Imaging Center Antwerp, University of Antwerp, Campus Drie Eiken – UC**,** Universiteitsplein 1**,** 2610 Wilrijk. **E-mail:** steven.staelens@uantwerpen.be **Tel:** +32 3 265 2820; **Fax:** +32 3 265 2813

|  |  | **2 day pi** | | **4 day pi** | | **7 day pi** | |
| --- | --- | --- | --- | --- | --- | --- | --- |
| **Antibody** | **Method** | **WT** | **APPPS1-21** | **WT** | **APPPS1-21** | **WT** | **APPPS1-21** |
| [^89^Zr]-Df-Bz-JRF/AβN/25 | iTLC | 91.9 ± 2.85 | 93.96 ± 2.03 | 94.33 ± 2.6 | 93.88 ± 2.73 | 96.37 ± 1.76 | 94.73 ± 1.28 |
|  | HPLC | 86.86 | 87.57 | 87.34 | 83.43 | 90.81 | 92.63 |

**Supplementary Table 1.** Stability measures of [^89^Zr]-labeled anti-amyloid antibody at different time points after intravenous
