# Supplementary material for: In Vivo Amyloid-β Imaging in the APPPS1–21 Transgenic Mouse Model with a 89Zr-Labeled Monoclonal Antibody
Source: Front Aging Neurosci. 2016 Mar 31;8:67. doi: 10.3389/fnagi.2016.00067 (PMC4815004; doi:10.3389/fnagi.2016.00067)
Supplement: Supplementary file 2 [file Table_2.DOCX]

***Supplementary Material***

***In vivo* amyloid-β imaging in the APPPS1-21 transgenic mouse model with a ^89^Zr- labeled monoclonal antibody.**

Ann-Marie Waldron^1,2^, Jens Fissers^1^, Annemie van Eetveldt^2^, Bianca Van Broeck^3^, Marc Mercken^3^, Darrel J. Pemberton^3^, Pieter Van Der Veken^4^, Koen Augustyns^4^, Jurgen Joossens^4^, Sigrid Stroobants^5^, Stefanie Dedeurwaerdere^2^, Leonie wyffels^1,5^, Steven Staelens^1^*.

**Corresponding author:** Steven Staelens, Molecular Imaging Center Antwerp, University of Antwerp, Campus Drie Eiken – UC**,** Universiteitsplein 1**,** 2610 Wilrijk. **E-mail:** steven.staelens@uantwerpen.be **Tel:** +32 3 265 2820; **Fax:** +32 3 265 2813

| **Antibody** | **Animal** | **Blood** | **Heart** | **Lungs** | **Liver** | **Spleen** | **Kidneys** | **Muscle** | **Skull**  **Bone** |
| --- | --- | --- | --- | --- | --- | --- | --- | --- | --- |
| [^89^Zr]-Trastuzumab | WT | 6.42 ± 1.6 | 1.82 ± 0.77 | 2.66 ± 1.12 | 3.51 ± 1.39 | 4.49 ± 1.76 | 2.96 ± 0.6 | 0.41 ± 0.14 | 2.63 ± 1.76 |
|  | APPPS1-21 | 7.16 ± 2.22 | 2.26 ± 0.76 | 3.7 ± 0.3 | 6.79 ± 2.24 | 5.59 ± 1.34 | 3.08 ± 0.84 | 0.5 ± 0.19 | 2.61 ± 0.41 |

**Supplementary Table 2.** Selected biodistribution data of [^89^Zr]-Trastuzumab 4 days after intravenous injection in WT mice and APPPS1-21 mice. Data is represented as the average percent of injected activity per gram tissue ± standard deviation.
