# Supplementary material for: In Vivo Amyloid-β Imaging in the APPPS1–21 Transgenic Mouse Model with a 89Zr-Labeled Monoclonal Antibody
Source: Front Aging Neurosci. 2016 Mar 31;8:67. doi: 10.3389/fnagi.2016.00067 (PMC4815004; doi:10.3389/fnagi.2016.00067)
Supplement: Supplementary file 4 [file Table_4.DOCX]

***Supplementary Material***

***In vivo* amyloid-β imaging in the APPPS1-21 transgenic mouse model with a ^89^Zr- labeled monoclonal antibody.**

Ann-Marie Waldron^1,2^, Jens Fissers^1^, Annemie van Eetveldt^2^, Bianca Van Broeck^3^, Marc Mercken^3^, Darrel J. Pemberton^3^, Pieter Van Der Veken^4^, Koen Augustyns^4^, Jurgen Joossens^4^, Sigrid Stroobants^5^, Stefanie Dedeurwaerdere^2^, Leonie wyffels^1,5^, Steven Staelens^1^*.

**Corresponding author:** Steven Staelens, Molecular Imaging Center Antwerp, University of Antwerp, Campus Drie Eiken – UC**,** Universiteitsplein 1**,** 2610 Wilrijk. **E-mail:** steven.staelens@uantwerpen.be **Tel:** +32 3 265 2820; **Fax:** +32 3 265 2813

**Supplementary Figure 1.** Comparison of brain retention of [^89^Zr]-Df-Bz-JRF/AβN/25 and [^89^Zr]-trastuzumab in WT and APPPS1-21 mice at 4 day p.i as measured by γ-counting.
